# Pregnancy and Delivery in Ehlers-Danlos Syndrome (Hypermobility Type): Review of the Literature

**DOI:** 10.1155/2011/306413

**Published:** 2011-06-15

**Authors:** Indranil Dutta, Helen Wilson, Odiri Oteri

**Affiliations:** Department of Obstetrics & Gynaecology, Lincoln County Hospital, Greetwell Road, Lincoln LN2 5QY, UK

## Abstract

Ehlers-Danlos syndrome (EDS) is a group of connective tissue disorders which are divided into various distinguishable phenotypes. The type of EDS determines the potential obstetric complications. Due to the spectrum of clinical manifestation and overlap between phenotypes, there are no standardised obstetric management guidelines. Existing literature illustrates different obstetric management in hypermobility type of EDS, including uneventful term vaginal deliveries as well as preterm cesarean section deliveries. This paper discusses obstetric management of a woman with EDS hypermobility type. Cesarean section was deemed the most appropriate delivery method in this patient due to the possible complications including risk of joint dislocation and pain morbidity. No obstetric complications were experienced, and good maternal and neonatal outcomes were achieved.

## 1. Introduction

The first comprehensive clinical description of Ehlers-Danlos Syndrome (EDS) was completed by Dr. Tschernogobow in 1892. The syndrome derives its name from a Danish dermatologist, Edward Ehlers in 1901, and a French physician and dermatologist, Henri-Alexandre Danlos in 1908. Later, clinical diagnostic criteria for the types of EDS were proposed by Beighton in 1998. EDS is a rare inherited disorder of connective tissue, characterised by a collagen synthesis defect. The group of related conditions share a common decrease in the tensile strength and integrity of the skin, joints, and other connective tissues. All forms of EDS share common features to varying degrees including joint hypermobility, skin hyper extensibility, tissue fragility, poor wound healing, and easy bruising [1]. 

Complications relating to EDS are infrequently seen in obstetric practice [2]. It presents with a range of considerations, which are specific to the classification of type. Some types are associated with severe maternal complications, whereas others are associated with more favourable outcomes [1]. There are at least six distinguishable phenotypes; however, overlap between these types is significant, making clinical diagnosis complex. The types include classic, hypermobility, vascular, kyphoscoliosis, arthrochalasia, and dermatosparaxis. Hypermobility type (formerly type III) is considered the least severe type of the syndrome, although important musculoskeletal complications can occur. It is inherited in an autosomal dominant pattern although the causative gene remains unidentified. Diagnosis of this type is based on clinical assessment alone. Major diagnostic criteria include joint hypermobility, skin involvement (smooth and velvety), and absence of fragility or other significant skin or soft tissue abnormalities (suggestive of other types of EDS) [3]. 

Recurrent joint dislocation, chronic joint pain, functional bowel disorders, and postural orthostatic tachycardia are features of minor diagnostic criteria. Obstetric management of EDS hypermobility type is largely specific to the individual, in particular musculoskeletal considerations. There is a continuing need to expand awareness regarding optimal management for EDS hypermobility type relating to antenatal, intrapartum, and postpartum management. 

## 2. Case Report

A 29-year-old woman was diagnosed with EDS hypermobility type at age 23. Retrospectively, her symptoms presented at age 14 years with low back pain, anterior knee pain, and multiple episodes of subluxation/dislocation of both hips. The patient also had easy bruising, papyraceous scars, early onset striae atrophicae, and irritable bowel syndrome. She suffered from chronic joint pain and received support from physiotherapists. Prior to her pregnancy, she had undergone multiple operations including tonsillectomy, appendectomy, meniscectomy of temporomandibular joint, a turbinectomy nasal operation at age 26 and colposcopy and LLETZ procedure due to severe dyskaryosis at age 27 (biopsy revealed CIN 3 and subsequent smears were normal). All except the nasal operation were uncomplicated procedures. Due to excessive bleeding following this operation, she received a transfusion of four units of blood. The bleeding settled spontaneously and no further intervention was required. There was no evidence of platelet dysfunction (which can sometimes be associated). She had previously consulted cardiologists following episodes of palpitations and associated tachycardia. Investigation with 24-hour tape showed daytime tachycardia which normalised at night, a feature of postural orthostatic tachycardia (POTS). She did not require further cardiology input or treatment for this. 

Family history comprised of early onset hip osteoarthritis of the patient's mother and maternal grandmother. No formal diagnosis of EDS had been given in any of the patient's family members. 

This was the first pregnancy of this patient. She was commenced under obstetric consultant led care following her dating scan at 13 weeks. Following this initial consultation, she was referred to a rheumatologist, haematologist, cardiologist, and anaesthetist. Rheumatology review stated no input was required although advice was given regarding keeping joints in neutral positions if under regional anaesthesia due to hypermobility and the risk of dislocation. Following consultation with haematology, no evidence of platelet dysfunction or increased risk of bleeding was identified. Cardiology input advised a preoperative ECG and echocardiography which were normal. However, she was not evaluated for classical EDS and genetic counselling was never offered. 

Obstetric plans included the need for prophylactic steroids at 28 weeks due to increased risk of premature rupture of membranes associated with EDS. She was booked for an elective cesarean section at 36 weeks, and if spontaneous labour occurred prior to this, she was to have an emergency cesarean section. The plan for cesarean section was due to the previous history of recurrent hip subluxation/dislocation in this case. Anaesthetic consultation prior to delivery included advice suggesting precise surgical haemostasis, and the availability of cross-matched blood prior to surgery due to potential fragility of blood vessel walls. The patient preferred to be awake and accepted the small risk of bleeding associated with spinal. Anaesthetic awareness of potential arrhythmias and/or hypertension during the operation was also noted. 

She was hospitalised at 24 weeks due to palpitations and shortness of breath. Pulmonary embolism was investigated by ventilation/perfusion (V/Q) scan which was negative. Symptoms settled without intervention. She had a small antepartum haemorrhage (APH) and tightening at 28 weeks and was admitted for observation. The patient was later discharged once reviewed by cardiology and her symptoms resolved, again, without intervention. Serial growth scans and cervical length monitoring were within normal limits and reassuring. 

At 35 weeks, the patient was admitted to delivery suite in spontaneous labour. She had an emergency lower segment cesarean section under spinal anaesthesia due to spontaneous onset of labour, as per delivery plan. Joints were maintained in neutral positions and no musculoskeletal problems occurred. No anaesthetic complications arose. Estimated blood loss was 400 mLs.The wound was closed with subcutaneous sutures, and wound healing was uncomplicated. Good postoperative recovery was made. Delivery of a healthy male infant weighing 1920 grams was achieved with Apgar scores of 9 in 1 min and 9 in 5 mins. The baby was kept in SCBU (Special care baby unit) for 4 days due to prematurity, hypothermia, hypoglycaemia, and for establishment of feeding. 

## 3. Discussion

In assessing potential complications that may occur in the antenatal, intrapartum, and postnatal periods in a patient with EDS, the type and severity of EDS should be identified. The subsequent implications for obstetric management can then be understood in accordance with the type-specific considerations. Understanding of the types of EDS and their relevant complications presented in obstetric care has developed in recent years, and there is now an emphasis in current literature to report type-specific issues, an aspect previously criticized [4]. Hypermobility type of EDS is associated with relatively benign musculoskeletal problems including joint dislocation and pain. No contraindications to pregnancy in this type of EDS have been described. Conversely classical and vascular types of EDS can have serious implications in pregnancy, and so prenatal counselling is vital for these patients [5, 6]. 

The incidence of EDS has been estimated and ranges between 1 in 5,000 and 1 in 20,000. The incidence of all types of EDS in pregnancy is estimated at 1 in 15,000 [7]. Classical and hypermobility types are the most common types of EDS, accounting for 60% of all EDS. Classical type (formally types I and II) has association with skin and soft tissue fragility, haemorrhage and poor wound healing. The vascular type (formally type IV) accounts for just 10% of all cases. It carries a high risk of maternal morbidity and mortality [8] which is estimated to be as high as 25% [6], predominantly due to spontaneous arterial rupture [9–11]. The other types of EDS are rarer [12]. 

Reported cases of obstetric outcomes in hypermobility-type EDS detail different delivery methods with good reported outcomes in all. One case outlines a patient with knee instability and nerve root pain from a prolapsed intervertebral disk. Following admission to hospital due to severe back pain at 27 weeks, cesarean section was undertaken at 35 weeks due to unretractable pain [1, 13]. Further case reports of hypermobility type EDS reported two patients with this diagnosis, both achieved term vaginal deliveries and neither of which experienced significant problems attributable to EDS [1, 4]. The authors suggest that hypermobility-type EDS can result in uneventful pregnancies without an increase in musculoskeletal pain and successful vaginal deliveries. This case was not without pregnancy complications; however, none of these complications were associated with the musculoskeletal problems anticipated in this type of EDS. The small APH at 28 weeks did not lead to any further bleeding, which was a concern, especially considering her previous history of excessive bleeding following nasal surgery. Preterm spontaneous labour is associated with EDS [12], and premature rupture of membranes leading to premature births in EDS has been well documented [1, 14]. The cause for preterm labour in this case could not be identified. In this case, cervical length was closely monitored and within normal limits. No other potential complications associated with all types of EDS, including delayed wound healing and postpartum haemorrhage, occurred. 

## 4. Conclusion

There are no obstetric management guidelines for patients with EDS, which reflects the wide range of potential type-specific implications, as well as the range of severity within these types [15]. Pregnancies in patients with EDS hypermobility type, as illustrated by this case, can be well tolerated and with good outcomes following cesarean section delivery. This suggests obstetric management plans should be made on a case-by-case basis, taking into account the diagnosis of type and severity of EDS, to optimise maternal and neonatal outcomes. 
